# SARS-CoV-2, the Angiotensin Converting Enzyme 2 (ACE2) Receptor and Alzheimer’s disease

**Published:** 2021-05-10

**Authors:** Walter J. Lukiw

**Affiliations:** 1 LSU Neuroscience Center, Louisiana State University Health Science Center, New Orleans LA 70112 USA; 2 Department of Neurology, LSU Neuroscience Center Louisiana State University Health Science Center, New Orleans LA 70112 USA; 3 Department of Ophthalmology, LSU Neuroscience Center Louisiana State University Health Science Center, New Orleans LA 70112 USA

## Description

Coronavirus disease of the year 2019 (COVID-19) is categorized as an acute, rapid onset viral pneumonia caused by the novel severe acute respiratory syndrome coronavirus 2 (SARS-CoV-2). SARS-CoV-2 is a member of the *Betacoronavirus* genus in the family *Coronaviridae* closely related to the severe acute respiratory syndrome coronavirus (SARS-CoV) and the Middle East Respiratory Syndrome coronavirus (MERS-CoV). Of all coronaviruses SARS-CoV-2 has emerged as an exceedingly pathogenic, highly transmissible lethal virus currently causing a serious pandemic of global proportions. The World Health Organization (WHO) reports that there are currently about ~141 million cases of COVID-19 in 219 countries along with about ~3.1 million total deaths as of mid-April 2021 [1-3]. SARS-CoV-2 mainly infects surface epithelial cells of the alveoli located in the lower respiratory tract of humans, causing acute lung injury, severe pneumonia and acute respiratory distress syndrome resulting in high morbidity and mortality. However due to the ubiquity of the ACE2 receptor many other cell and tissue types and physiological systems are also involved in COVID-19 infection, and this may in part explain the extensive variety of the signs and symptoms observed in COVID-19 patients [3]. Interestingly, the zoonotic emergence of SARS-CoV-2 and the role of intermediate hosts such as old world fruit bats (*Pteropus scapulatus*) are considered one of the major natural mammalian repositories of SARS-CoV-2 and the probable source of interspecies transmission [4-7].

SARS-CoV-2 is an unusually large, enveloped *Betacoronavirus* containing a positive sense, single stranded RNA (ssRNA) genome of about ~29,811 nucleotides (nt) that encodes multiple membrane proteins including the spike protein (S1) essential for SARS-CoV-2-cell fusion and viral entry into human host cells [8,9]. The SARS-CoV2 RNA virus is therefore considerably considerably larger than the size of the average cellular messenger RNA (mRNA; size range ~2,000-5,000 nt). SARS-CoV-2 invasion of, and replication within, susceptible human host cells is a complex process that initially requires an S1-mediated viral protein interaction with the angiotensin-converting enzyme 2 (ACE2) type 1 transmembrane receptor located on the surface of multiple epithelial and endothelial cells of the respiratory system, and a large number of both immune and nonimmune cell types. Normally, the primary physiological role of the dipeptidyl carboxydipeptidase ACE2 receptor, an 805 amino acid, 92.5 kDa, zinc-containing membrane-integral metalloprotein (E.C.3.4.17.23) is in the binding and maturation of angiotensin, a circulating peptide hormone that functions as a vasodilator, controls vasoconstriction, regulates blood flow and blood pressure in cardiovascular and renal function, modulates ischemia in the cardio-vasculature and neurovasculature and serves as a facilitator of amino acid transport, representing multiple aspects of pathophysiology that are impacted in the Alzheimer’s disease (AD) brain [10,11]. The ACE2 receptor is not only found on the outer surface of many different types of human respiratory cells but is also abundant in the majority of cell types of the brain, CNS and visual system [12-14]. In fact in part due to the ubiquity of the ACE2 surface receptor SARS-CoV-2 has a remarkable and unusual capacity to attack many different types of human host cells simultaneously, exploiting any immune weakness in the host, and as such is deleterious to diverse multiple host cell types, tissues and organ systems at the same time [8,15,16]. Interestingly the highest ACE2 expression found to date in the human brain and CNS is in the pons and medulla oblongata of the brainstem, containing the brain’s medullary respiratory centers, and this may in part explain the susceptibility of many SARS-CoV-2 infected patients to experience severe respiratory distress [12,17-19].

**Figure 1:**
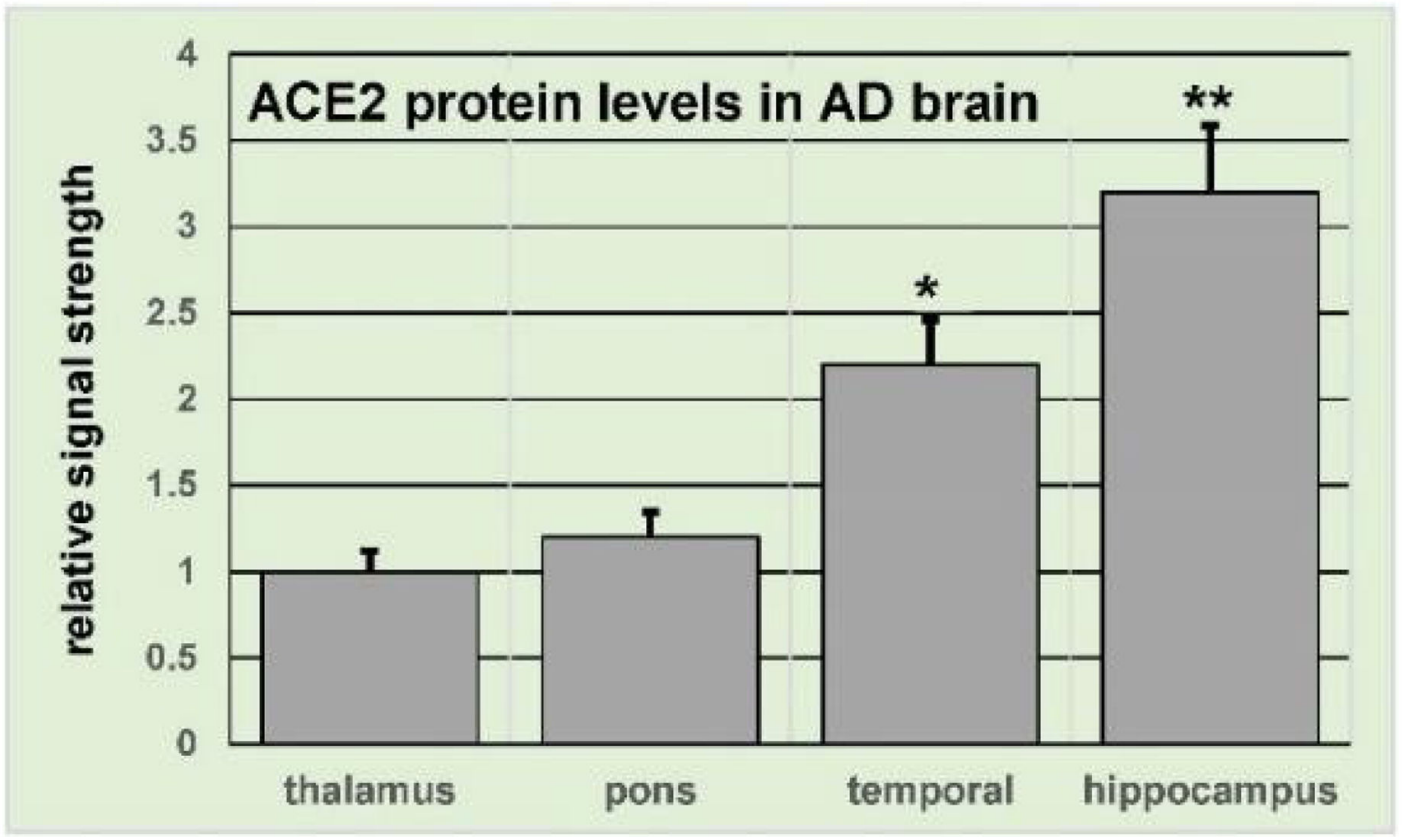
ACE2 expression (at the level of ACE2 protein) is increased in selected AD brain regions. Tissue-specific patterns of ACE2 expression at the protein level in human brain thalamus, pons, temporal lobe association neocortex (Brodmann area A22) and hippocampus were determined using a human ACE2 ELISA Kit (ab235649; Abcam, Cambridge MA, USA) in AD versus age-and gender-matched elderly controls; the control group (N=3) had a mean age of 75.5 ± 12.7 years and a mean post-mortem interval (PMI; death to brain-freezing period) of ~3.5 hours; the AD group (N=3) had a mean age of 76.1 ± 11.4 years and a mean post-mortem (PMI;) of ~3.4 hours; all brain samples were from female donors; there was no significant difference in the mean age, gender, PMI, yield or purity of total RNA between the control and the AD groups; in control human brain the pons, containing the medullary respiratory centers exhibits the highest concentration of ACE2 receptors of 21 brain regions analyzed [12; see text]; no significant difference in ACE2 receptor expression (at the level of protein) was found between control or AD thalamus and in this brain region the relative signal strength was set to 1.0; the pons was found to have an ACE2 expression of 1.15 AD over control which was not significant; on the other hand the temporal lobe neocortex and hippocampus exhibited 2.2-fold and 3.2-fold increases in ACE2 expression respectively; the temporal lobe association neocortex and hippocampus are targeted neuroanatomical regions in AD neuropathology; all results are represented as relative signal strength which is defined as fold-change increases in AD over control; N=3 control or N=3 AD brain tissue samples were used for each determination; a minimum of N=3 ELISA analyses were performed for each protein determination in tissues; *p<0.05; **p<0.01 (ANOVA); error bars represent one standard deviation of the mean.

Multiple laboratories including our own have reported that in several brain regions ACE2 expression is significantly higher in patients with AD than in age and gender-matched controls both at the level of increased ACE2 mRNA [11,12] and protein [20]; manuscript in preparation] (Figure 1). Ascertaining whether the mechanism of the upregulation of ACE2 expression involves oxidative stress and whether increased SARS- CoV-2 promotes oxidative stress is suspected but requires further investigation [11,12,20]. Data is emerging that aged individuals with pre-existing neurological conditions, and especially African Americans and women appear to represent a distinctive human population subset with both a significantly increased incidence of COVID-19 with a higher likelihood of adverse clinical outcomes [21-23]. In two recent comprehensive analytical studies of ~62 million adult patients from 360 hospitals and 317,000 providers across all 50 US states indicated patients with neurological disorders including depression, schizophrenia and AD had a significantly increased risk for COVID-19 infection from 2 to 8-fold over controls [8,21,22]. In these mixed COVID-19-dementia cases common presenting clinical features were found to include confusion and delirium (82.4%), asthenia (76.8%), fever (72.8%), polypnea (51.2%) and low blood oxygen (desaturation; 50.4%), and a persistent ‘brain fog’, confusion, mood and behavioral disorders were observed in survivors (19.2%) [24,25]. As a consequence of the disease AD patients: (i) typically exhibit a down-regulation of personal hygiene with increased forgetfulness; (ii) may forget to wear a face mask or wear a face mask incorrectly; (iii) forget to wash their hands as often as they should or wash their hands ineffectively; and (iv) may inadvertently expose themselves to over-populated situations such as crowds-all factors which significantly increase their risk for acquiring SARS-CoV-2 infection and COVID-19. Thus, from multiple epidemiological perspectives and based on both molecular-genetic and/or behavioral criteria patients with AD are at risk of being highly affected by the COVID-19 pandemic [26].

The abundant release of cytokines and a dysfunctional immune response in COVID-19 patients leads to a profound surge in the mobilization of immune cells and hyper-inflammation, triggering additional massive cytokine and chemokine release sometimes referred to as the ‘cytokine storm’ [19,27]. As a result, COVID-19 patients typically exhibit higher levels of pro-inflammatory, modulatory cytokines such as TNFα, INFγ, IL-1β, IL-2, IL-4, IL-6, IL-7, IL-9, IL-10, IL-12, IL-13, IL-17, G-CSF, GM-CSF, MCSF, HGF and chemokines such as CXCL8, MCP1, IP10, MIP1α and MIP1β [19,27]; these same increases in pro-inflammatory cytokines and chemokines are often observed in the brains of AD patients [11,20,26]. Complications associated with the cytokine storm include severe respiratory distress, intravascular blood coagulation, multi-organ failure, cardiovascular anomalies, disrupted innate-immune responses, neurovascular complications and neuroinflammation [19,26,27]. A ‘smoldering’ neuroinflammation and inflammatory neurodegeneration are prominent features of progressive, age-related neurodegenerative disorders and appear to play a major role in the neuropathology of AD with higher susceptibility to more severe long-term pathological outcomes after infection by SARS-CoV-2 [19,26,27].

Lastly, emerging research evidence continues to suggest a significant mechanistic overlap between AD and COVID-19, strongly centered on SARS-CoV-2 invasion via the natural ubiquity of ACE2 receptors, neurovascular inflammation and brain injury [11,12,20]. Like many viruses SARS-CoV-2 is strongly neurotropic tending to attack or affect the structure and function of neurons and support cells of the human brain and central and peripheral nervous systems (CNS, PNS). Results from multiple independent research laboratories: (i) continues to warrant the continuing testing and careful monitoring of AD patients with COVID-19 for a possible higher SARS-CoV-2 viral load in respiratory fluids, the systemic circulation, the brain and CNS and long-term adverse neurological consequences in aging patients with dementia; and (ii) highlight the need to protect and vaccinate demented patients including those with AD as part of the overall strategy to gain control over the current COVID-19 pandemic.

## Significance of the Research

A wealth of current research data [25 of the 27 (92.6%) of the references quoted in this Commentary are from the publication years 2020 or 2021] continues to strengthen the molecular-genetic link between COVID-19 and lethal, progressive, age-related neurological diseases that include AD; the essential cell surface receptor ACE2 for initial host cell binding of SARS-CoV-2 is providing a valuable link in furthering our understanding of the pathological mechanism of AD and the potential involvement of SARS-CoV-2.
